# Lobophorin C and D, New Kijanimicin Derivatives from a Marine Sponge-Associated Actinomycetal Strain AZS17

**DOI:** 10.3390/md9030359

**Published:** 2011-03-17

**Authors:** Rong-Bian Wei, Tao Xi, Jing Li, Ping Wang, Fu-Chao Li, Yong-Cheng Lin, Song Qin

**Affiliations:** 1 College of Marine Science, Zhejiang Ocean University, Zhoushan 316004, China; E-Mails: windswingcn@yahoo.com.cn (R.-B.W.); wp77319@163.com (P.W.); 2 Marine Drugs Research Centre, China Pharmaceutical University, Nanjing 210018, China; E-Mail: xitao@cpu.edu.cn; 3 Open Key Laboratory of Experimental Marine Biology, Institute of Oceanology, Chinese Academy of Sciences, Qingdao 266071, China; E-Mail: lifuchao@ms.qdio.ac.cn; 4 School of Chemistry and Chemical Engineering, Sun Yat-sen University, Guangzhou 510275, China; E-Mails: zsulijing@163.com (J.L.); ceslyc@mail.sysu.edu.cn (Y.-C.L.)

**Keywords:** marine sponge, actinomycete, lobophorin C and D, cytotoxic activity

## Abstract

Marine sponge *Hymeniacidon* sp. was collected from coastal waters of the East China Sea to isolate symbiotic microorganisms. The resulting sponge-associated actinomycete, *Streptomyces carnosus* strain AZS17, was cultivated in a 20 L volume of medium for production of bioactive secondary metabolites. Bioassay-guided isolation and purification by varied chromatographic methods yielded two new compounds of kijanimicin derivatives, AS7-2 and AS9-12. Their structures were elucidated by spectroscopy and comparison with literatures. Results showed these two compounds were structurally similar to the previously reported compounds lobophorin A and B, yet differed in specific bond forms, stereochemistry and optical activities. The two novel compounds were named lobophorin C and D. *In vitro* cytotoxicity investigation by MTT assay indicated their selective activities. Lobophorin C displayed potent cytotoxic activity against the human liver cancer cell line 7402, while lobophorin D showed significant inhibitory effect on human breast cancer cells MDA-MB 435.

## 1. Introduction

Sponges (Porifera) remain the most important phylum in the field of marine drugs discovery, since they produce a great number of novel natural products with a variety of potent pharmacological activities. Nevertheless, the transformation of marine sponge-derived active compounds into drugs has been hindered by supply limitation. It is well known that sponges are hosts for a large amount of microorganisms, demonstrating interactions such as epibiotic, symbiotic and parasitic relationships, *etc.* Symbiotic functions that have been attributed to marine sponge microbial associates include nutrient acquisition and secondary metabolite production [1]. The various secondary metabolites synthesized by microbial associates inhabiting marine sponges also possess good bioactivities in many studies [2–6]. In recent years, microbial diversity in the marine environment, particularly marine sponge-associated microorganisms, have become a significant source of new lead compounds for marine drugs with vast biotechnological potential.

During the course of our investigation for bioactive natural products from marine sponge-associated microorganisms, we have isolated some known metabolites from marine sponge-derived fungi. In this paper, we report the isolation and characterization of two new compounds of kijanimicin derivatives from the fermentation broth of a variant strain of *Streptomyces carnosus* associated with marine sponges *Hymeniacidon* sp. The *in vitro* cytotoxic activities were detected against two human cancer cell lines. This is the first report of bioactive metabolites derived from *Streptomyces* associated with marine sponges of the genus *Hymeniacidon* according to the current review of Thomas [7].

## 2. Results and Discussion

**Figure 1.**
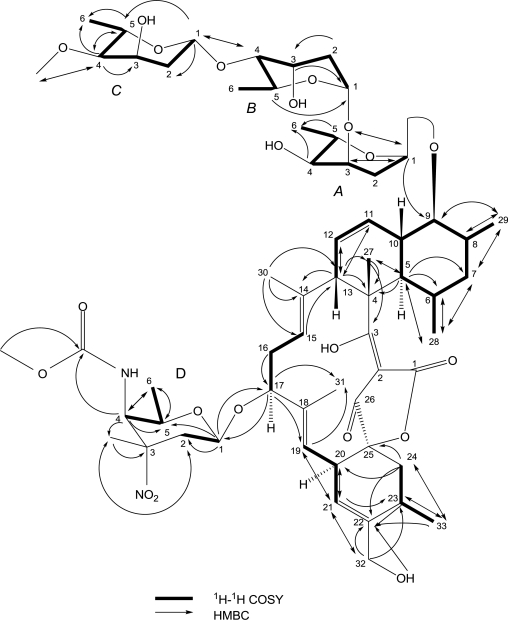
The key ^1^H-^1^HCOSY and HMBC correlations of compound **1**.

Compound **1** (lobophorin C) was obtained as a white amorphous powder, soluble in dimethyl sulphoxide (DMSO) and methanol. It gave the [M + Na]^+^ ion at *m/z* 1209 in the ESI-MS, indicating a molecular weight of 1186. HR-ESI-MS analysis displayed the [M + Na]^+^ signal at *m/z* 1209.5958 (calculated 1209.5934). Element analysis revealed the compound consists of N (2.019%), C (54.11%), H (7.219%), O (36.65%). Thus, the molecular formula of AS7-2 was established to be C_61_H_90_N_2_O_21_ based on HR-ESIMS, ^1^H and ^13^C NMR data (Table 1) and element analysis, with 18 sites of unsaturation. The IR spectrum of **1** showed bands characteristic of hydroxyl (3432 cm^−1^), ester carbonyl (1721 cm^−1^) groups and olefinic bonds (1631 cm^−1^). UV spectrum exhibited the maximum absorption bands at 267 and 241 nm. The ^1^H-NMR spectrum demonstrated the presence of eleven methyl protons, two methoxyl protons (δ 3.59, s; 3.26, s), one anomeric proton and a NH proton signal (δ 7.40). The ^13^C-NMR spectrum of **1** (Table 1) revealed signals for 61 carbons. ^13^C-NMR and various DEPT spectra allowed the assignments of the 61 carbon signals to eleven methyl, two methoxyl (δ 55.7, 51.9), eight methylene and 29 methine and eleven quaternary carbon atoms. Among them, four anomeric carbons (δ 99.08, 97.63, 97.34, 91.20) suggested the presence of four sugar residues. The correlation of the adjoining proton resonances was investigated by ^1^H-^1^H COSY, showing the HH connectivities as: H_15_-H_16_-H_17_, H_19_-H_20_-H_21_, H_24_-H_23_-H_33_, H_13_-H_12_-H_11_-H_10_-H_9_-H_8_-H_29_, H_8_-H_7_-H_6_ and H_10_-H_5_-H_6_-H_28_ (Figure 1). The correlation of neighboring protons in A-ring sugar moiety was found to be H_1A_-H_2A_-H_3A_-H_4A_-H_5A_-H_6A_, similar proton connectivities in B-ring and C-ring sugar moieties can be derived from the cross peaks of the ^1^H-^1^H COSY spectrum. In the HMBC experiment, the following key correlations were observed in the aglycone part of compound **1**: H-5 to C-7, C-6, C-4, C-28; H-7 to C-29, C-28; H-8 to C-29; H-13 to C-27, C-14, C-12, C-11, C-4; H-21 to C-19, C-20, C-23, C-32; H-24 to C-33, C-25, C-22, C-20; H-27 to C-5, C-4, C-3; H-28 to C-7, C-6, C-5; H-29 to C-9, C-8, C-7; H-30 to C-15, C-14, C-13; H-32 to C-23, C-22, C-21; H-33 to C-24, C-23, C-22 (Table 1 and Figure 1). HMBC correlations were detected within the sugar units as: H-6_D_ with C-5_D_, C-4_D_; H-5_C_ with C-6_C_, C-4_C_; H-4_C_ with C-5_C_, C-3_C_, C-6_C_; H-2_B_ with C-3_B_; H-3_B_ with C-1_B_; H-5_A_ with C-6_A_, C-1_A_; H-4_A_ with C-6_A_; H-1_A_ with C-3_A_, C-5_A_ (Table 1 and Figure 1). The points of attachment for the sugar moieties of ring A and ring D to the C-9 and C-17 sites of the aglycone, respectively, were determined via the long-range correlations of H-1_A_ to C-9, H-1_D_ to C-17, as well as H-17 to C-1_D_.

**Figure 2.**
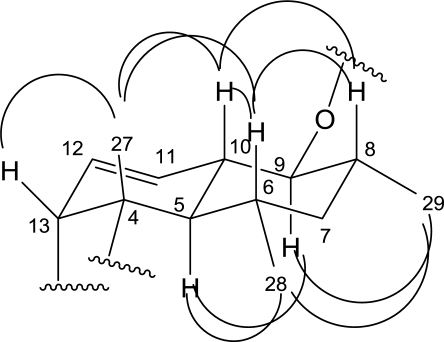
The key NOESY correlations of compound **1**.

The NOESY spectrum provided information regarding the relative configuration of the compound **1**. Key NOE interactions were recorded between H-5 (δ 2.00) and H-9 (δ 3.29), H-5 and H-28 (δ 0.55) as shown in Figure 2, implying H-9, H-5 and the methyl group in site 28 (H-28) were close to each other with same orientation. Also, NOE interaction can be observed between H-28 and H-29 (δ 1.01), indicating the orientation of the CH_3_ group in site 28 was identical with the CH_3_ group in site 29. Accordingly, they should be in the same side of the aglycone plane.

**Figure 3.**
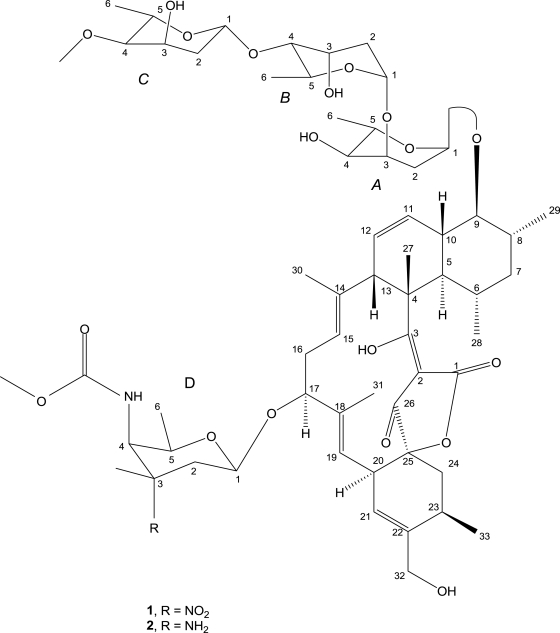
The structures of lobophorin C (**1**) and D (**2**).

The structure of compound **1** was very similar to the compound lobophorin B, derived from an unidentified actinomycete strain CNC-837 isolated from the surface of the Caribbean brown alga *Lobophora variegata* [8]. Both of them were derivatives of the terrestrial antibiotic kijanimicin [9], with one less sugar moiety than kijanimicin and contained one 2,6-dideoxy-4-*O*-methyl-l-ribo-hexopyranose and two 2,6-dideoxy-l-ribo-hexopyranose in their sugar units. By comparison of their spectral data, compound **1** is distinctly different from the known lobophorin B in these aspects: Firstly, a –OH group and a C=O group linked to the sites of C-3 and C-26 in compound **1** respectively, while a C=O and a –OH attached to these positions in lobophorin B, respectively. Consequently, the unsaturated double bond between C-3 and C-2 and the saturated single bond between C-2 and C-26 in compound **1** were substituted by a saturated bond and an unsaturated double bond in lobophorin B, respectively, exhibiting transformations between enol and ketone forms. These changes can be confirmed by chemical shifts in the corresponding positions (Table 1). Secondly, the two CH_3_ groups in C-28 and C-29, which were in opposite orientation in lobophorin B, were in the same side in compound **1** by NOE analysis. Besides, the optical rotation of compound **1** was −150.9°, in contrast with +104° of lobophorin B when measured in same condition. To sum up, the structure of compound **1**, which derived from a marine sponge symbiotic actinomycete, was not identical to the previously reported compound lobophorin B due to mutual enol and ketone transformations in local groups, relative stereochemistry and optical activities. Therefore, compound **1** was named lobophorin C. Its structure is shown in Figure 3.

Compound **2** (lobophorin D) was obtained as a white amorphous powder like lobophorin C (**1**). The molecular weight was deduced as 1156 from the pseudomolecular ions [M + H]^+^ at *m/z* 1157 in positive ESI-MS and [M − H]^−^ at *m/z* 1155 in negative ESI-MS. Element analysis showed the compound consist of N (2.049%), C (50.10%), H (7.103%), O (40.75%). The molecular formula of compound **2** was determined as C_61_H_92_N_2_O_19_ based on ESI-MS, ^1^H and ^13^C NMR spectra and element constitution analysis. ^13^C NMR and DEPT spectra displayed 11 CH_3_ carbon signals, 2 –OCH_3_ carbons; 8 CH_2_ carbons and 29 CH carbons. HSQC spectrum revealed 11 quaternary carbons without protons attachment. Compound **2** resembled lobophorin C (**1**) in signals assignments by ^1^H-^1^HCOSY coupled with HMBC spectra. Both showed great structural similarities except that the substitute group in C-3 of sugar ring D was –NH_2_ in compound **2** instead of –NO_2_ in lobophorin C (**1**). Compound **2** exhibited almost identical structure with the published compound lobophorin A, except that a hydroxyl group and a carbonyl group linked to the sites of C-3 and C-26 in the aglycone part of compound **2**, respectively, deduced by its spectral NMR data. In addition, both the methyl groups of C-29 and C-28 in compound **2** were in the same spatial orientation, and compound **2** displayed an optical rotation of −138.4° while −175° was observed for lobophorin A. Hence, compound **2** was named lobophorin D (Figure 3).

**Figure 4.**
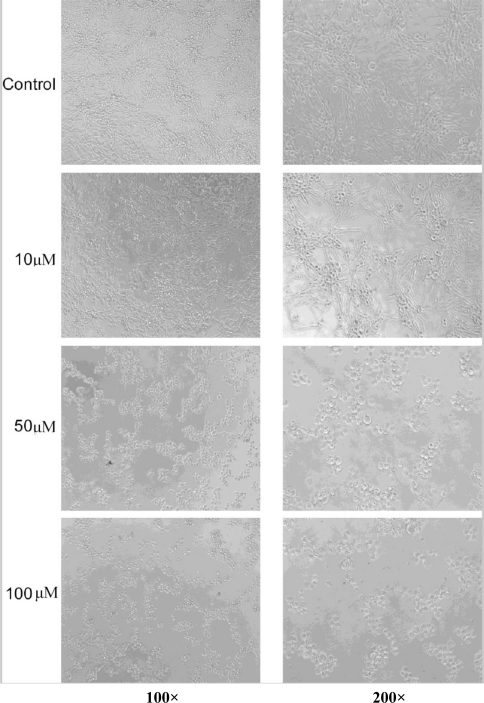
Effect of compound **2** on the growth of the human breast cancer cell line MDA-MB 435 under different concentrations.

The cytotoxicity of compounds **1** and **2** were tested *in vitro* against a human liver cancer cell line 7402 and a human breast cancer cell line MDA-MB 435. The results showed compound **1** demonstrated a strong cytotoxic activity while compound **2** was inactive against the cell proliferation of 7402 hepatoma cells, with IC_50_ values of 0.6 μg/mL and 723.1 μg/mL, respectively. On the contrary, compound **2** displayed a good inhibitory effect on the growth of human breast cancer cells MDA-MB 435 with an IC_50_ value of 7.5 μM, in contrast to the negligible activity of 61.8 μM for compound **1**. Apoptosis of the breast adenocarcinoma cells can be observed with increasing concentrations of compound **2** (Figure 4). Compounds **1** and **2** seemed to have selective cytotoxicity for these cancer cell lines. Though as derivatives of kijanimicin with similar structures, compounds **1** and **2**, in contrast with lobophorin A and B demonstrated different bioactivities. The previously reported lobophorins A and B did not exhibit significant antibiotic properties, whereas they showed potent anti-inflammatory activities. It is suggested that the distinction of bioactivities was likely attributed to the differences of stereochemistry of the two groups of compounds and their optical activities, as well as the slight modification of specific bonds. The mechanism of cytotoxic selectivity of compounds **1** and **2** to various cancer cell lines needs to be further investigated.

## 3. Experimental Section

### 3.1. General Procedures

Optical rotations were measured on a Horiba High Sensitivity Polarimeter SEPA-300. UV and IR spectra were measured on a Shimadzu UV-2501PC spectrophotometer and a Bruker VECTOR 22 FT-IR spectrophotometer, respectively. NMR spectroscopic data were recorded on a Varian INOVA 500NB NMR spectrometer. ESIMS and HR-ESIMS spectra were measured on a Thremo LCQ DECA XP LC mass spectrometer and a Q-TOF Ultima Global GAA076 LC mass spectrometer respectively. Element analysis used an Elementar Vario EL CHNSO elemental analyzer.

### 3.2. Strain Isolation and Fermentation

Actinomycete strain AZS 17 was isolated as an endosymbiotic microorganism from a marine sponge collected from the intertidal zone of Qingbang island of Zhoushan Archipelago in the East China Sea. The sponge was identified as *Hymeniacidon* sp.—which is likely to be a new species within the genus *Hymeniacidon*—by Professor Jinhe Li, Institute of Oceanology, Chinese Academy of Sciences. The actinomycetic strain was preliminary identified as a *Streptomyces* species based on morphology observation and physio-biochemical characteristics. Molecular sequencing of the 16S rRNA gene (Accession number FJ999670) revealed it was a variant species of *Streptomyces carnosus*.

Strain AZS 17 cultivated on Gause’s I slant medium was streaked onto a agar plate containing a half seawater-based medium (malt extract 10 g, yeast extract 4 g, glucose 4 g and agar 18 g dissolved in 0.5 L artificial seawater and 0.5 L tap water for sterilization and solidification) and cultured at 28 °C for 4 days in an incubator. The acquired seed culture was cut into small pieces before a small piece was inoculated into a 1000 mL Erlenmeyer flask containing 250 mL of the above culture medium without agar in a total volume of 20 L, subsequently incubated in a shaker under 28 °C, 110 rpm for 5 days.

### 3.3. Extraction and Isolation

The fermented culture (20 L) was filtered through kieselguhr (Sinopharm Reagent, Shanghai, China) to separate the mycelia. The filtrate was extracted with ethyl acetate three times, while the mycelia were treated under ultrasonication before extraction by EtOAc. The combined extracts were evaporated *in vacuo* at 37 °C to yield 7.89 g brown oily residue. This crude extract was chromatographed on a silica gel column using a stepwise gradient of petroleum ether–EtOAc–MeOH to afford nine fractions. Brine shrimp bioassay-guided fractionation excludes others to target fr.7 and fr.9. Fr.7 was subjected to Sephadex LH-20 column chromatography eluting with CHCl_2_–MeOH (1:1) to obtain 4 fractions. Fr.7-2 was selected to reverse phase column chromatography on a YMC (YMC Co. Ltd., Japan) ODS column. Preparative thin layer chromatography with Merck precoated TLC plate and repeated Sephadex LH-20 column chromatography purification yielded compound **1** (51.3 mg). Similar methods were applied to the isolation of Fr.9 and eventually obtained compound **2** (42.6 mg).

### 3.4. Cytotoxicity Assays and Observation

Human liver cancer cells 7402 were obtained from the Cell Bank of Type Culture Collection of Shanghai Institute of Cell Biology, the Chinese Academy of Sciences. Human breast adenocarcinoma cells MDA-MB 435 were provided by Cancer Research Centre, Sun Yat-sen University, China. Cells were maintained in RPMI-1640 supplemented with 10% fetal bovine serum, 100 U/mL penicillin, and 100 mg/mL streptomycin sulfate at 37 °C, 5% CO_2_.The MTT assay was performed as described previously [10]. The IC_50_ values were calculated by SPSS software with Probit model. The morphological changes were observed under an inverted microscope at different magnifications. Photos were taken after 24 h of drug treatment before MTT dyeing.

## Figures and Tables

**Table 1.** NMR data of compound **1** (*δ* in ppm, *J* in Hz).

**No.**	**^13^C**	**^1^H**	**HMBC**	**^1^H-^1^H COSY**	**NOESY**
1	173.6 C				
2	96.5 C				
3	197.4 C				
4	50.4 C				
5	43.4 CH	2.00(m,overlap *)	C-28,27,7,6,4	H-6,10	H-28,9
6	31.1 CH	1.38(m,overlap)	C-28	H-28	H-27,10,8
7	41.7 CH_2_	1.51(m,overlap), 1.38(m,overlap)	C-29,28	H-8,6	
8	34.1 CH	2.09(m,overlap)	C-29	H-29	H-10,6
9	84.0 CH	3.29(m,overlap)	C-29	H-10,8	H-29,5
10	38.5 CH	1.89(m,overlap)		H-11,9,5	H-27,8,6
11	125.2 CH	5.65(d,10.5)	C-13	H-12	
12	128.0 CH	5.28(m,overlap)	C-13	H-13,11	
13	50.1 CH	3.71(m,overlap)	C-27,14,12,11,4	H-12	H-27
14	136.0 C				
15	121.4 CH	5.06(brs)	C-13	H-16	
16	35.1 CH_2_	2.06(m,overlap)	C-17	H-17	
17	79.1 CH	4.07(d,5.6)	C: 31,19, D-1	H-16	
18	135.1 C				
19	120.4 CH	5.02(d,8)	C-31,21	H-20	
20	39.5 CH	3.21(d,8)	C-21	H-21,19	
21	123.3 CH	5.31(s)	C-32,23,20,19	H-20	
22	140.6 C				
23	27.1 CH	2.45(m,overlap)	C-33,22	H-33,24	
24	35.5 CH_2_	2.48(m,overlap) 1.43(m,overlap)	C-33,25,22,20	H-23	
25	91.1 C				
26	199.9 C				
27	14.8 CH_3_	1.38(s)	C-5,4,3		H-13,10,6
28	22.3 CH_3_	0.55(d,4.4)	C-7,6,5	H-6	H-29,5
29	13.9 CH_3_	1.01(d,5.6)	C-9,8,7	H-8	H-28,9
30	15.0 CH_3_	1.41(s)	C-15,14,13		
31	14.0 CH_3_	1.26(s)			
32	63.5 CH_2_	4.02(m,overlap) 3.88(m,overlap)	C-23,22,21	OH-4.60	
33	19.8 CH_3_	1.16(d,5.6)	C-24,23,22	H-23	
A-2	29.2 CH_2_	2.21(brd,12) 1.65(brd,12)		H: A-2, A-1	
A-3	66.9 CH	3.85(m,overlap)	C: A-1	H: A-4, A-2	
A-4	70.8 CH	3.15(m,overlap)	C: A-6	H: A-5, A-3, OH4.79	
A-5	64.0 CH	3.96(m,overlap)	C: A-6, A-1	H: A-6, A-4	
A-6	17.4 CH_3_	1.09(d,5.2)		H: A-5	
B-1	91.2 CH	5.05(brs)		H: B-2	
B-2	35.2 CH_2_	1.85(m,overlap)	C: B-3	H: B-3, B-1	
B-3	66.2 CH	3.91(brs)	C: B-1	H: B-2, B-4	
B-4	81.4 CH	3.14(m,overlap)	C: C-1	H: B-3, B-5	
B-5	61.9 CH	3.96(m,overlap)	C: B-1	H: B-4, B-6	
B-6	17.9 CH_3_	1.10(d,5.2)		H: B-5	
C-1	99.1 CH	4.82(d,7.6)	C: C-5, C-2, B-4	H: C-2	
C-2	37.8 CH_2_	1.54(m,overlap)		H: C-3, C-1	
C-3	61.9 CH	4.15(brs)		H: C-4, C-2, OH4.60	
C-4	82.1 CH	2.76(dd,9.5,2.5)	C: C-6, C-5, C-3, C-4OCH_3_	H: C-5, C-3	
C-5	67.5 CH	3.69(m,overlap)	C: C-6, C-4	H: C-6, C-4	
C-6	18.2 CH_3_	1.13(d,5.2)		H: C-5	
C-4OCH_3_	55.7 CH_3_	3.26(s)	C: C-4		
D-1	97.6 CH	4.36(d,7.6)	C: 17, D-5, D-2	H: D-2	
D-2	30.8 CH_2_	2.27(m,overlap) 2.08(m,overlap)	C: D-3CH_3_	H: D-2, D-1	
D-3	82.0 C				
D-4	53.1 CH	4.24(d,8.4)	C: D-6,D-5, D-4C=O, D-3CH_3_	H: D-5, NH	
D-5	68.6 CH	3.43(m,overlap)	C: D-6	H: D-6, D-4	
D-6	16.8 CH_3_	1.01(d,5.2)	C: D-5, D-4	H: D-5	
D-3CH_3_	24.9 CH_3_	1.43(s)	C: D-3, D-2		
D-4C=O	157.7 C				
D-4OCH_3_	51.9 CH_3_	3.59(s)	C: D-4		

The spectra were recorded in DMSO-d6 at 500 MHz for ^1^H and 125 MHz for ^13^C;

* Due to signal overlapping, coupling constants could not be determined.
